# Impact of patient choice and hospital competition on patient outcomes after rectal cancer surgery: A national population‐based study

**DOI:** 10.1002/cncr.34504

**Published:** 2022-10-19

**Authors:** Lu Han, Jemma M. Boyle, Kate Walker, Angela Kuryba, Michael S. Braun, Nicola Fearnhead, David Jayne, Richard Sullivan, Jan van der Meulen, Ajay Aggarwal

**Affiliations:** ^1^ Department of Health Services Research and Policy London School of Hygiene and Tropical Medicine London UK; ^2^ Clinical Effectiveness Unit Royal College of Surgeons of England London UK; ^3^ Department of Oncology The Christie NHS Foundation Trust Manchester UK; ^4^ School of Medical Sciences University of Manchester Manchester UK; ^5^ Department of Colorectal Surgery Cambridge University Hospitals Cambridge UK; ^6^ University of Leeds Leeds UK; ^7^ Institute of Cancer Policy King’s College London London UK; ^8^ Department of Oncology Guy’s & St. Thomas’ NHS Trust London UK

**Keywords:** cancer surgery, health care markets, hospital competition, patient choice, patient outcomes

## Abstract

**Background:**

The objective of the current national cohort study was to analyze the correlation between choice and competition on outcomes after cancer surgery in rectal cancer.

**Methods:**

The analysis included all men who underwent rectal cancer surgery in the English National Health Service between March 2015 and April 2019 (*n* = 13,996). Multilevel logistic regression was used to assess the effect of a rectal cancer surgery center being located in a competitive environment (based on the number of centers within a threshold distance) and being a successful competitor (based on the ability to attract patients from other hospitals) on eight patient‐level outcomes: 30‐ and 90‐day emergency readmissions, 30‐day re‐operation rates, 90‐day postoperative mortality, length of stay >14 days, circumferential resection margin status, rates of primary procedure with a permanent stoma, and rates of persistent stoma 18 months after anterior resection.

**Results:**

With adjustment for patient characteristics, patients who underwent surgery in centers located in a stronger competitive environment were less likely to have an abdominoperineal excision or a Hartman's procedure (odds ratio [OR], 0.73; 95% confidence interval [CI], 0.55–0.97, *p* = .04). Additionally, individuals who received treatment at hospitals that were successful competitors had a lower risk of a 90‐day readmission following rectal cancer surgery (OR, 0.86; 95% CI, 0.76–0.97, *p* = .03) and were less likely to have a persistent stoma at 18 months after anterior resection (OR, 0.75; 95% CI, 0.61–0.93, *p* = .02).

**Conclusions:**

Hospitals located in areas of high competition are associated with better patient outcomes and improved processes of care for rectal cancer surgery.

## Abstract 2

The results of this study build on an emerging evidence base that demonstrates that hospitals located in areas of high competition are associated with better care quality. These results continue to challenge centralization or regionalization of some cancer surgical services as the dominant approach to deliver improvements in quality.

## INTRODUCTION

Policies that encourage patient choice and hospital competition have been introduced in several countries with the objective of improving the efficiency and quality of health services.^1^, ^2^ However, the impact of hospital competition on outcomes of cancer treatment remains largely unknown.

In high‐income settings, previous research within prostate cancer surgery has demonstrated that men who underwent surgery in a competitive environment (based on the density of service provision within a specified geographical area) were less likely to have 30‐day emergency readmissions, regardless of the volume of procedures performed at each hospital.^3^ Another study demonstrated that women with breast cancer were more likely to receive immediate reconstruction after mastectomy if they were treated in the most competitive hospitals.^4^ However, there remains a lack of empirical evidence across different tumor types and treatment modalities.

There is an urgent need to understand better if hospital competition can act as a driver for outcome improvement given the present focus on increasing specialization and centralization of surgical care to fewer high volume hospitals to deliver improvements in outcome.^5^ This policy however is at odds with choice and competition because it limits the number of hospitals from which patients can choose and also market incentives for improvement in care quality.^6^, ^7^

Rectal cancer surgery is one such cancer where there is an increasing narrative around the specialization of care^8^, ^9^, ^10^ due to the complexity of the surgery and the management pathway that continues to be refined (e.g., watch‐and‐wait strategies, multimodal treatments).^11^, ^12^ However, the literature to date remains equivocal as to evidence for a volume outcome relationship, although some guidelines do not recommend treatment in low volume centers (<10 per annum per hospital).^13^

In this study, we use national patient‐level data on all patients who underwent rectal cancer surgery in the English National Health Service (NHS) between April 2015 and March 2019 to analyze whether hospitals located in a competitive environment and those that are successful competitors (i.e., hospitals that attract patients from other hospitals) have better patient‐level outcomes.

The English NHS is a model health system for studies aiming to get a better understanding of the implications of patient choice and hospital competition policies. First, it is a publicly funded health care system covering the entire population enabling inclusion of a national sample of patients from different regions that are characterized by variations in the configuration of services. Second, there is empirical evidence that patients in the NHS are responsive to patient choice policies.^14^, ^15^ Third, patient outcomes following rectal cancer treatment are publicly reported at the individual hospital level.^16^

## MATERIALS AND METHODS

Hospital admission records from the Hospital Episode Statistics (HES), the administrative database that contains a record of all hospital episodes in the English NHS, were obtained on all patients diagnosed with rectal cancer who underwent a major resection between April 1, 2015 and March 31, 2019 within the English NHS. Patients were included in our analysis if they had undergone elective major rectal cancer surgery (including anterior resection, Hartmann's procedure, abdominoperineal resection [APR], panproctocolectomy, or pelvic exenteration) and if they were treated in the 163 pre‐identified NHS hospitals that routinely perform rectal cancer surgeries. Patients who were diagnosed with metastatic disease (M1) before surgery or those underwent surgery in the private sector (<10% of eligible patients) were not included in the analysis.

HES provided information on patient‐level characteristics including age on admission, sex, ethnicity, the number of comorbidities according to the Royal College of Surgeons Charlson comorbidity score,^17^ socioeconomic deprivation expressed in terms of quintiles of national distribution of the index of multiple deprivation (IMD), treating surgical hospital, diagnoses according to International Classification of Diseases, 10th edition (ICD‐10) codes, surgical procedures coded according to Office of Population Censuses and Surveys Classification of Surgical Operations and Procedures, 4th revision (OPCS‐4), and the dates of these procedures. HES data were used to derive the average volume of procedures performed annually at each hospital.

Patients were electronically linked at patient level to records of the National Bowel Cancer Audit (NBOCA). The NBOCA is a prospective mandatory database for all patients newly diagnosed with colorectal cancer in the English NHS.^16^ This provided additional information on each patient's disease status and treatment, including date of diagnosis, cancer stage (tumor, node, metastasis [TNM]), American Society of Anesthesiologists (ASA) score, and Eastern Cooperative Oncology Group performance status. It also provides information on postoperative outcomes as outlined below. Linkage with the radiotherapy data set enabled identification of patients receiving preoperative radiotherapy.

### Patient outcomes

In this study, we created eight patient‐level outcome measures using our linked NHS data sets. The choice of outcome measures were based on those used by the National Institute for Health and Care Excellence (NICE), the national body that publishes clinical guidelines in England and Wales, when they assessed the association of volume and outcomes for rectal cancer^13^ as well as recent work by Fokas and colleagues^18^ when they explored the advantages and disadvantages of different end points in rectal cancer.

#### Length of stay

Data items from HES records were used to calculate the duration of inpatient stay from the date of a major rectal resection. A binary outcome was created to indicate an inpatient stay beyond 14 days.

#### 30‐ And 90‐day emergency readmissions

HES records were used to identify patients readmitted as an emergency at any NHS hospital within 30 or 90 days from the date of discharge following a major rectal resection for any cause. Readmission rates have been used extensively as an outcome indicator for emergency and elective surgical admissions.^19^, ^20^, ^21^, ^22^

#### 30‐Day re‐operation rates

HES records were analyzed to identify patients who went back to theatre within 30 days for complications arising from the index major bowel resection. The measure captures complications such as anastomotic leak, bleeding, and infection necessitating a further surgical procedure and is derived from a set of validated HES OPCS codes.^23^ This is an important measure because it has been shown to impact morbidity, short‐, and long‐term mortality as well as oncological and functional outcomes.

#### 90‐Day mortality

Data items in HES records derived from information from the Office for National Statistics (ONS) were used to identify patients who died within 90 days of their rectal cancer surgery.

#### Circumferential resection margin status

Circumferential resection margin (CRM) status is a data item from the NBOCA and is an established measure of surgical quality. A positive CRM is associated with higher rates of local and distant recurrence.^24^

#### Primary procedure with a permanent stoma

We assessed the proportion of patients having a permanent stoma because of their primary rectal cancer procedure. This included any APR, panproctocolectomy, pelvic exenteration, or Hartmann’s procedure. This is important because the decision regarding which operation to perform considers several different factors including the location (height from anal verge) and staging of the tumor, comorbidities, likely functional outcome, response to neoadjuvant therapy, patient preference, and surgical experience/skill. The wider literature has demonstrated that disparities in stoma formation may also reflect socio‐demographic and geographic differences across populations.^25^

#### Persistent stoma at 18 months after anterior resection

We assessed the proportion of patients undergoing anterior resection who had a stoma remaining at 18 months. Most stomas formed during an anterior resection are temporary and expected to be reversed.^26^, ^27^ A persistent stoma at 18 months may be the result of complications both intraoperatively and postoperatively, as well as factors precluding reversal such as surgical capacity.

### Hospital characteristics

Two hospital‐level factors were considered in this analysis that has previously been validated for this type of analysis.^3^ The first was the hospitals' “competitive environment” that reflects the number of hospitals and the number of patients within the catchment area of each hospital. The second is the extent to which hospitals are “successful competitors” derived from their ability to attract patients from other hospitals.

#### Competitive environment

**FIGURE 1:**
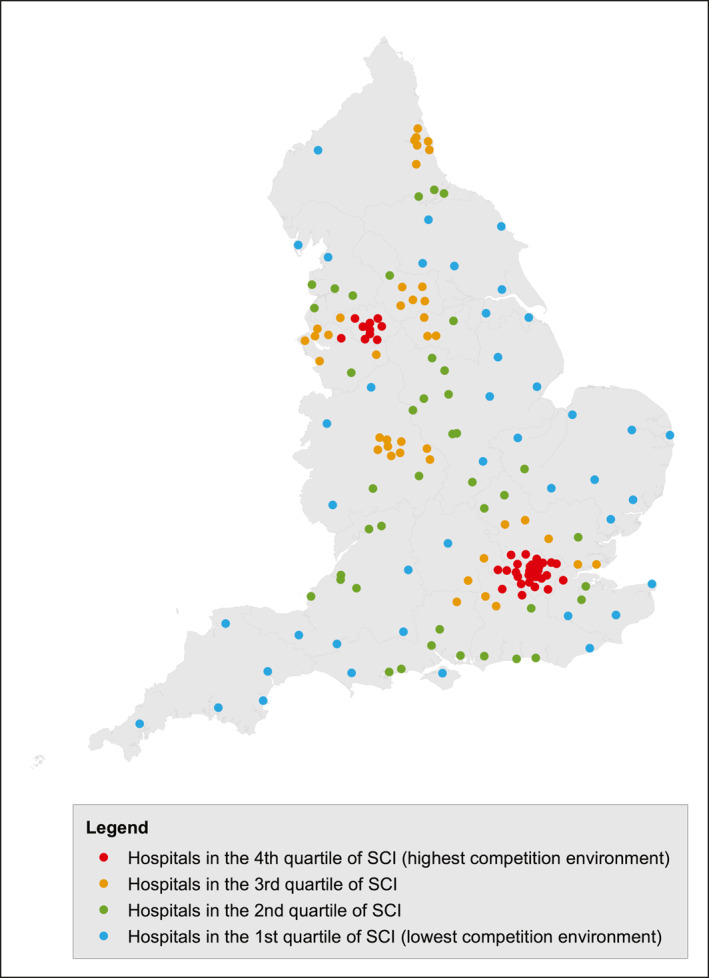
Map of England demonstrating the geographical distribution of hospitals providing rectal cancer surgery according to the level of competition in their area. See Materials and Methods for further explanation.

For each hospital offering rectal cancer surgery, we calculated a spatial competition index (SCI)^28^, ^29^ based on the number of eligible patients within a 30‐minute drive time from each hospital (the catchment area of a hospital) and the number of alternative surgical hospitals within 30‐minute drive for each eligible patient:

$${SCI}_{i}=1-\frac{1}{n_{i}}\sum_{j_{i}=1}^{n_{i}}\frac{1}{k_{j_{i}}},$$

where hospital “*i*” has *n* eligible patients within a 30‐minute drive and patient *j* in hospital *i* has *k* alternative surgical hospitals within a 30‐minute drive. The spatial competition index ranges theoretically from 0 for a hospital where every patient in its catchment area has only one hospital available within a 30‐minute drive time, up to a value close to 1 where patients in its catchment area have a large number of hospitals available within a 30‐minute drive time. Hospitals were grouped into quartiles according to the strength of the competition they faced and their geographical distribution across the United Kingdom is demonstrated in Figure 1.

#### Successful competition

Using established methods,^14^ we identified hospitals that were “successful” or “unsuccessful” competitors. For each hospital, we identified two groups of patients: (1) “leavers”—patients for whom that hospital was nearest in terms of drive times, but who had their treatment elsewhere, and (2) “arrivers”—patients for whom another surgical provider was nearest but who had their surgery at that hospital. Rectal cancer surgical hospitals were subsequently stratified into three groups based on whether they were successful competitors (having a statistically significantly [*p* < .05] higher number of arrivers than leavers), unsuccessful competitors (having a statistically significantly higher number of leavers than arrivers), or having a statistically insignificant difference between arrivers and leavers. We used the conditional method for testing a difference between two Poisson means to determine whether the differences between arrivers and leavers were statistically significant.^30^, ^31^

### Statistical analysis

Multilevel logistic regression analyses with a random intercept at hospital level were used to assess the effect of receiving surgery in hospitals located in a competitive environment and being a successful competitor on the eight patient outcome indicators with adjustment for the patient case‐mix (age, sex, ethnicity, socioeconomic status, cancer stage, comorbidity status, performance status, ASA grade, receipt of radiotherapy, and year of treatment) and hospital‐level procedure volume. All statistical analyses were undertaken in Stata version 15.

## RESULTS

**FIGURE 2:**
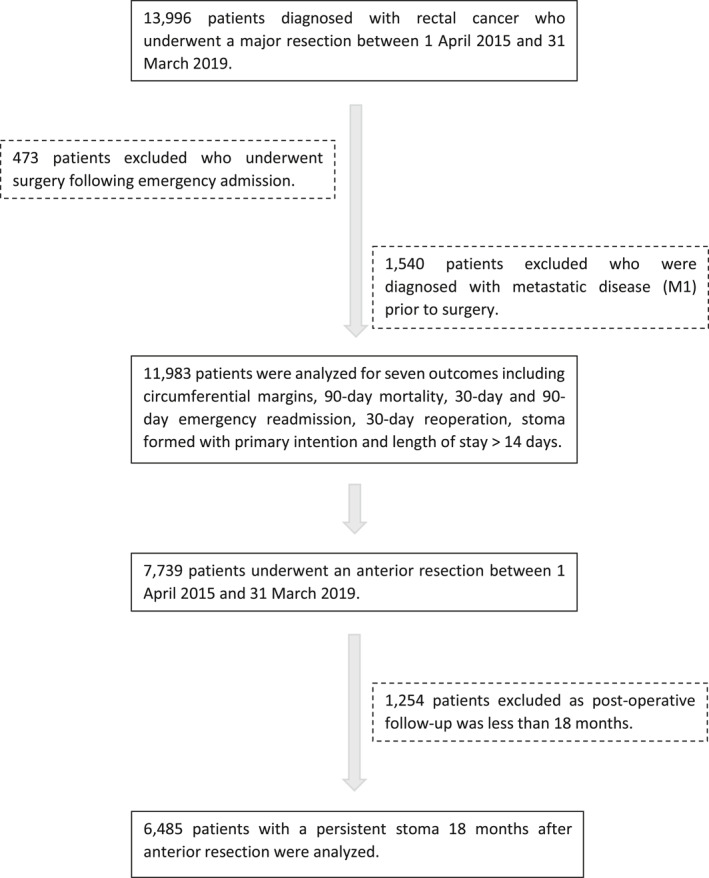
Flow diagram for patient inclusion in the analysis cohort.

We identified 13,996 patients who underwent a major resection for rectal cancer between April 1, 2015 and March 31, 2019 in the English NHS. A total of 473 were excluded because they had emergency surgery. A total of 1540 were excluded because they had been diagnosed with metastatic disease (M1) before the index surgery. A total of 11,983 patients were included in the analyses (Figure 2).

Table 1 presents the characteristics of the patients in the overall cohort and of a subsample of patients who underwent an anterior resection. The mean age of the overall study cohort was 67, of which 65.0% were male and 41.0% had one or more comorbidity. Table S1 stratifies the overall cohort according to the spatial competition index of their treating hospital and Table S2 according to whether they were treated in a hospital which was a successful or unsuccessful competitor. Table 2 demonstrates the incidence of the different outcome measures across the overall cohort and the variation across surgical hospitals. For example, the average rate of primary stoma forming rectal cancer surgery was 34.3% with variation between 0% and 86.5% across the 163 hospitals that perform rectal cancer surgery in the English NHS. The incidence of 90‐day postoperative death was 1.6% with a variation of 0% to 10.0% across the hospitals.

**TABLE 1:** Patient characteristics

	Patients who underwent a major rectal cancer resection		Patients who underwent an anterior resection only	
	No.	%	No.	%
No. of patients, *N*	11,983	100	6485	100
Age, mean (SD), years	67 (11.2)		66 (11.0)	
<50	839	7.0	458	7.1
50–59	2128	17.8	1196	18.4
60–74	5988	50.0	3381	52.1
75–84	2659	22.2	1303	20.1
≥85	369	3.1	147	2.3
Sex				
Male	7805	65.1	4201	64.8
Female	4178	34.9	2284	35.2
Ethnicity				
White	10,841	90.5	5853	90.3
Mixed	537	4.5	317	4.9
Missing	605	5.1	315	4.9
IMD quintiles				
1st (least deprived)	1777	14.8	912	14.1
2nd	2061	17.2	1064	16.4
3rd	2570	21.5	1391	21.5
4th	2739	22.9	1496	23.1
5th	2813	23.5	1611	24.8
Missing	23	0.2	11	0.2
Charlson comorbidities				
0	7066	59.0	3989	61.5
1	3387	28.3	1752	27.0
≥2	1530	12.8	744	11.5
ASA grade				
1	1760	14.7	1088	16.8
2	7061	58.9	3897	60.1
≥3	2631	22.0	1222	18.8
Missing	531	4.4	278	4.3
Performance status				
0 (normal activity)	6925	57.8	3819	58.9
1 (walk and light work)	2904	24.2	1472	22.7
2+ (walk and all self‐care: up >50%)	857	7.2	417	6.4
Missing	1297	10.8	777	12.0
T staging				
T1	1538	12.8	858	13.2
T2	3416	28.5	1828	28.2
T3	5551	46.3	3085	47.6
T4	680	5.7	341	5.3
Missing	798	6.7	373	5.8
N staging				
N0	7325	61.1	3944	60.8
N1	2726	22.8	1518	23.4
N2	1134	9.5	647	10.0
Missing	798	6.7	376	5.8
M staging				
M0	11,167	93.2	6098	94.0
M1	105	0.9	57	0.9
Missing	711	5.9	330	5.1
Preoperative radiotherapy				
No preoperative treatment reported	7977	66.6	4792	73.9
Long course RT presurgery	3115	26.0	1270	19.6
Short course RT presurgery	891	7.4	423	6.5
Year of surgery				
2016	2903	24.2	1826	28.2
2017	2967	24.8	1835	28.3
2018	3173	26.5	1940	29.9
2019	2940	24.5	884	13.6
Spatial competition index^a^				
1st quartile (range, 0–0.44, 41 sites)	3350	28.0	1721	26.5
2nd quartile (range, 0.44–0.73, 41 sites)	3598	30.0	2029	31.3
3rd quartile (range, 0.73–0.87, 41 sites)	3198	26.7	1739	26.8
4th quartile (range, 0.87–0.95, 40 sites)	1837	15.3	996	15.4
Successful or unsuccessful competitors^b^				
Successful (49 sites)	4314	36.0	2335	36.0
Unsuccessful (56 sites)	3403	28.4	1881	29.0
Hospitals with no significant gain or loss of patients (58 sites)	4266	35.6	2269	35.0
Volume				
1st tertile, low (60 sites)	2367	19.8	1305	20.1
2nd tertile, medium (51 sites)	3607	30.1	1948	30.0
3rd tertile, high (55 sites)	6009	50.2	3232	49.8

Abbreviations: ASA, American Society of Anesthesiologists; IMD, index of multiple deprivation; RT, radiotherapy; SCI, spatial competition index; SD, standard deviation.

^a^ SCI is a hospital‐based measure assessing the level of competition between hospitals within a 30‐minute drive time. This has been categorized into four quartiles based on the level of competition. 1st quartile represent hospitals in the lowest competition areas and 4th quartile represents hospitals in the highest competition areas.

^b^ Successful competitors are centers that have a statistically significant net gain of patients and Unsuccessful competitors are centers that had a statistically significant net loss of patients (see Materials and Methods).

**TABLE 2:** Patient outcomes

	*n*	% (range across hospitals)
No. of patients, *N*	11,983	100
90‐day postoperative death		
No	11,787	98.4
Yes	196	1.6 (0–10.0)
Circumferential margins		
No	9294	77.6
Yes	814	6.8 (0–47.5)
Missing	1875	15.7
30‐day readmission		
No	10,301	86.0
Yes	1682	14.0 (0–50.0)
90‐day readmission		
No	8908	74.3
Yes	3075	25.7 (9.5–50.0)
30‐day reoperation		
No	10,601	88.5
Yes	1382	11.5 (0–50.0)
Stoma (primary intention)^a^		
No	7872	65.7
Yes	4111	34.3 (0–86.5)
LoS >14 days		
No	9506	79.3
Yes	2442	20.4 (0–52.0)
Missing	35	0.3
Stoma at 18 months^b^	6485	100
No	4740	73.1
Yes	1745	26.9 (0–69.2)

Abbreviations: APR, abdominoperineal resection; LoS, length of stay.

^a^ This included patients who had an APR, Hartmann’s, or pelvic exenteration (see Materials and Methods).

^b^ This included patients who underwent anterior resection with a follow‐up of at least 18 months (see Materials and Methods).

A total of 6485 patients within our cohort underwent an anterior resection (Table 1). This subsample is presented separately because it allowed us to assess the proportion of patients in this group who had a stoma present at 18 months following their procedure. The proportion of patients with a stoma 18 months following their anterior resection was 26.9% with variation between 0% and 69.2% across hospitals (Table 2).

### Competitive environment

Table 3 presents the results from the multilevel logistic regression model as adjusted odds ratios (OR) for the impact of a hospital being in a competitive environment on patient outcomes. Patients who underwent major rectal cancer surgery at hospitals located within areas with a strong competitive environment (2nd, 3rd, and 4th SCI quartiles) were at considerably lower risk (adjusted OR, 0.73; 95% confidence interval [CI], 0.55–0.97; overall *p* = .04) to receive primary surgery with permanent stoma formation than in hospitals located in the least competitive environment (1st SCI quartile [overall *p* = .038] after adjusting for patient characteristics). This effect on permanent stoma formation was present even when adjusting for the volume of procedures performed at each hospital (OR, 0.78; 95% CI, 0.58–1.05; overall *p* = .046) (Table S3). The strength of the competitive environment did not have a significant impact on any of the other seven outcome measures including persistent stoma at 18 months after anterior resection.

**TABLE 3:** Association between the competitiveness of the hospital environment, measured by the SCI on patient outcomes

	90‐Day death		Circumferential margins		30‐Day readmission		90‐Day readmission	
	OR^a^ (95% CI)	*p*	OR^b^ (95% CI)	*p*	OR^c^ (95% CI)	*p*	OR^d^ (95% CI)	*p*
SCI^a^ 1st quartile	Ref		Ref		Ref		Ref	
SCI 2nd quartile	0.82 (0.56–1.20)	.65	0.91 (0.60–1.38)	.94	0.95 (0.79–1.15)	.6	1.02 (0.88–1.18)	.87
SCI 3rd quartile	0.92 (0.61–1.39)		0.90 (0.60–1.35)		1.03 (0.85–1.25)		1.03 (0.90–1.18)	
SCI 4th quartile	1.07 (0.67–1.71)		0.98 (0.66–1.44)		0.89 (0.70–1.14)		0.96 (0.81–1.14)	

	30‐Day reoperation		Stoma (primary intention)^c^		Stoma (at 18 months)^d^		LoS >14 days	
SCI 1st quartile	Ref		Ref		Ref		Ref	
SCI 2nd quartile	1.04 (0.83–1.31)	.73	0.78 (0.61–0.99)	.04	1.07 (0.83–1.39)	.4	1.08 (0.88–1.32)	.1
SCI 3rd quartile	1.03 (0.79–1.36)		0.76 (0.60–0.95)		1.21 (0.92–1.60)		1.01 (0.82–1.25)	
SCI 4th quartile	0.91 (0.70–1.18)		0.73 (0.55–0.97)		0.97 (0.69–1.34)		1.30 (1.03–1.65)	

Abbreviations: APR, abdominoperineal resection; ASA, American Society of Anesthesiologists; CI, confidence interval; IMD, index of multiple deprivation; LoS, length of stay; OR, odds ratio; SCI, spatial competition index; TNM, tumor, node, metastasis.

^a^ OR adjusted for patient‐level characteristics (age, sex, ethnicity, IMD, Charlson comorbidities, ASA grade, performance status, TNM staging, preoperative radiotherapy, and year of surgery).

^b^ SCI is a hospital‐based measure assessing the level of competition between hospitals within a 30‐min drive time. This has been categorized into four quartiles based on the level of competition. 1st quartile represent hospitals in the lowest competition areas and 4th quartile represents hospitals in the highest competition areas.

^c^ This included patients who had an APR, Hartmann’s, or pelvic exenteration (see Materials and Methods).

^d^ This included patients who underwent anterior resection with a follow‐up of at least 18 months (see Materials and Methods).

### Successful competition

In total, 49 hospitals were identified as successful competitors and 56 were identified as unsuccessful competitors. For 55 hospitals, the difference between arrivers and leavers was not statistically significant (see Materials and Methods).

Table 4 presents the results from the multilevel logistic regression model. With adjustment for patient characteristics, individuals who received treatment at hospitals that were successful competitors were at a lower risk to have a 90‐day readmission following rectal cancer surgery compared to hospitals that were unsuccessful competitors (OR, 0.86; 95% CI, 0.76–0.97; *p* = .03). This effect was hardly affected by further adjustment for the volume of procedures performed at each hospital (OR, 0.85; 95% CI, 0.74–0.97; *p* = .02) (Table S4). In addition, patients treated at hospitals that were successful competitors were at considerably lower risk to have a persistent stoma at 18 months after anterior resection (OR, 0.75; 95% CI, 0.61–0.93; *p* = .02). This effect remained statistically significant when adjusted for volume of procedures (OR, 0.71; 95% CI, 0.58–0.88; *p* = .002) (Table S5). With respect to the other five outcome indicators, being a successful competitor did not have any significant association.

**TABLE 4:** Association between whether a hospital was a successful or unsuccessful competitor on patient outcomes

	90‐Day death		Circumferential margins		30‐Day readmission		90‐Day readmission	
	OR^a^ (95% CI)	*p*	OR^b^ (95% CI)	*p*	OR^c^ (95% CI)	*p*	OR^d^ (95% CI)	*p*
Unsuccessful competitors	Ref		Ref		Ref		Ref	
Successful competitors	0.89 (0.60–1.32)	.34	0.94 (0.65–1.38)	.51	0.86 (0.73–1.02)	.11	0.86 (0.76–0.97)	.03
Hospitals with no significant gain or loss of patients	1.16 (0.83–1.62)		0.83 (0.59–1.16)		0.86 (0.73–1.01)		0.88 (0.78–0.99)	

	30‐Day reoperation		Stoma (primary intension)^c^		Stoma (at 18 months)^d^		LoS >14 days	
Unsuccessful competitors	Ref		Ref		Ref		Ref	
Successful competitors	1.04 (0.84–1.28)	.93	1.10 (0.85–1.41)	.74	0.75 (0.61–0.93)	.02	1.03 (0.85–1.25)	.94
Hospitals with no significant gain or loss of patients	1.03 (0.85–1.26)		1.01 (0.81–1.27)		0.96 (0.75–1.23)		1.00 (0.85–1.19)	

Abbreviations: APR, abdominoperineal resection; ASA, American Society of Anesthesiologists; CI, confidence interval; IMD, index of multiple deprivation; LoS, length of stay; OR, odds ratio; TNM, tumor, node, metastasis.

^a^ OR adjusted for patient‐level characteristics (age, sex, ethnicity, IMD, Charlson comorbidities, ASA grade, performance status, TNM staging, preoperative radiotherapy, and year of surgery).

^b^ Successful competitors are centers that have a statistically significant net gain of patients and unsuccessful competitors are centers that had a statistically significant net loss of patients (see Materials and Methods).

^c^ This included patients who had an APR, Hartmann’s, or pelvic exenteration (see Materials and Methods).

^d^ This included patients who underwent anterior resection with a follow‐up of at least 18 months (see Materials and Methods).

Table S6 demonstrates the differences in average annual volume of procedures for hospitals in areas with a high or low competition, as well between successful competitors, unsuccessful competitors, and hospitals which did not have statistical evidence of a net gain or loss of patients. Hospitals located in areas of strongest competition (4th quartile) performed on average fewer procedures (*n* = 20) compared to hospitals located in areas of lower competition (*n* = 31; *p* < .001). Successful competitors performed on average a higher number of surgical procedures per annum (*n* = 35) compared to hospitals that lost patients to other hospitals (*n* = 22) or compared to those with no net gain or loss (*n* = 29; *p* < .001).

## DISCUSSION

Patients who underwent a major resection for rectal cancer at UK NHS hospitals located within areas with a strong competitive environment were less likely to have an abdominoperineal or Hartmann’s procedure than patients treated in areas where the competition between hospitals is less strong. In addition, patients treated at successful competitors (hospitals that attracted more patients from the catchment areas of other hospitals) were less likely to have a re‐admission within 90 days of their surgery and also less likely to have a persistent stoma 18 months after an anterior resection, compared with those who received treatment at hospitals that were unsuccessful competitors.

Our results build on an emerging evidence base that demonstrates that hospitals located in areas of high competition are associated with better care quality. This includes better patient outcomes,^3^, ^32^ improved processes of care (e.g., use of techniques associated with better outcomes or experience of care),^4^ and better availability of services such as the increased availability of new technologies, services, or staff.^29^ These results continue to challenge centralization or regionalization of some cancer surgical services as the dominant approach to deliver improvements in quality.

It is not certain what the drivers are for improved quality of care at hospitals located in the most competitive areas without further qualitative investigation, but our findings suggest it is not driven by a volume effect. A study by Bloom et al.^33^ demonstrated that hospitals located in areas with a stronger competitive environment had enhanced system and management practices.

Another potential driver of quality is the competition between hospitals for local patients. In competitive areas with a larger selection of available providers, patients are more likely to choose an alternative hospital based on their preferences or those of their primary care practitioner. As a result, hospitals seek to improve their care in order to retain and attract new patients. This may explain the finding that patients treated in hospitals located in the most competitive environments were more likely to undergo sphincter‐sparing surgery.^34^, ^35^, ^36^ Another plausible explanation is that there is simply greater access (due to a greater choice of hospitals) to surgeons or hospitals with the necessary expertise and experience of sphincter sparing low anterior resections in more competitive areas compared to less competitive areas, which have fewer hospitals available to choose from.

A further explanation is that both training and practices of care (e.g., adoption of robotic surgery^29^) may evolve more rapidly in hospitals located in more competitive environments compared to hospitals that are relatively isolated geographically. As trainees take up senior surgical posts within each region, practices of care become embedded with the evolution of care and are much more sensitive to knowledge transfer within these close networks. Similar evolution of practices has been observed in prostate cancer, where hospitals located in the most competitive areas were among the first adopters of robotic surgery.^29^ A US‐based study demonstrated greater uptake of mastectomy and reconstruction techniques in hospitals located in regions with high market competition.^4^

From a hospital perspective, we found that successful competitors had evidence of better patient outcomes, with fewer 90‐day emergency readmissions and a greater likelihood of having a stoma reversal following an anterior resection. These findings are important as NBCOA has publicly reported results at the hospital level on emergency readmissions and rates of stoma at 18 months since 2013. Our results therefore suggest that patients and their primary care practitioners may be responsive to public outcome reporting on these indicators as hospitals with the best outcomes in these domains, were preferentially selected by patients who were prepared to bypass their local hospitals to receive treatment there. This provides empirical evidence of the impact of hospital level reporting on patient hospital selection.^37^

The association between a successful competitor and a reduced hospital readmission rate at 90 days following surgery could reflect the experience and expertise of hospitals with respect to perioperative care as well as wider management processes. For example, improved outreach care following community discharge to avoid emergency readmissions. Other factors may include better patient selection for the procedure and excellence in pre‐rehabilitation that has been demonstrated to result in reduced complications and re‐admissions following surgery.^38^, ^39^

We also found evidence that patients treated at successful competitors were less likely to have a stoma at 18 months following an anterior resection. This may reflect three specific care characteristics. Fewer surgical or nonsurgical related complications following an anterior resection or adjuvant treatments, better patient selection through tumor review boards or MDTs, or higher operative and staff capacity to undertake timely reversals.

Our study relates to the NHS that is a single‐payer publicly funded system where prices are fixed and competition is expected to drive up quality. However, this type of analysis is also informative to international policy makers, particular in fixed or administered price managed health care markets such as those that have been implemented across Europe and the United States Medicare Program.^1^ This includes using our empirical approach to evaluate the impact of competition across a wide range of tumor types and interventions to support health service design.^4^ We consider two hospital‐based measures for competition. A measure that considers the level of potential competition among hospitals using the spatial competition index; and a measure, which measures the extent to which a given hospital is a successful competitor according to whether they gain patients from additional hospitals. We are also able to explore the effect of hospital volume on these associations.

In addition, this type of empirical analysis can help us evaluate whether systems that encourage a plurality of providers to support patient choice and competition are more likely to create both better access (due to increased availability of services) and quality, thereby offsetting inefficiencies resulting from hospitals not using all their available capacity nor benefit from economies of scale that occurs from hospital mergers.^40^ The framework could also support service centralization initiatives. For instance, one could consider consolidating services to centers that are preferentially selected (“successful competitors”) by patients and their primary care physicians.

There are a number of limitations of the present study. First, the spatial competition index is a proxy for actual competition between hospitals and there could potentially be misclassification of a small number of hospitals when stratifying centers according to the strength of competition they face within their areas. The findings are therefore hypothesis generating and the associations observed may relate to other factors that we cannot measure that may have influenced the decisions for treatment. Further embedded qualitative work is therefore required, in particular to get a better understanding of the impact of selective referral patterns and patterns of training and management in rectal cancer which may diffuse regionally. Second, further work is planned aiming to get a better understanding of the underlying differences in care delivery that can explain some of the observed associations, for example, the availability of minimally invasive surgery, greater utilization of neoadjuvant radiotherapy, postoperative chemotherapy, or integration of enhanced recovery after surgery protocols. Third, we have adjusted for a variety of patient characteristics, including socioeconomic status and ethnicity but there are likely to be unmeasured confounders that may influence these associations, for example, access to care givers. Fourth, some lower‐volume hospitals that are genuinely successful competitors will have been classified as nonsignificant competitors due to the lack of statistical power to demonstrate that they are successful competitors. Fifth, we acknowledge that the findings are unlikely to be broadly applicable to the low and middle income settings where health systems have mixed public and private systems and the degree of regulation of competition is variable.^41^ Sixth, we appreciate that there are further short‐term outcome measures that we were not able to assess, because they are not available within the routinely collected data sets used, for example, operative time and blood transfusions. The availability of data also precluded a more detailed evaluation of the reasons for readmission.

In conclusion, this national population‐based study provides timely evidence of an association between hospital competition and improvements in short and intermediate‐term outcomes following rectal cancer surgery. We also find that patients and referring primary care practitioners are responsive to the public reporting of measures of quality with preferential selection for hospitals with better outcomes, which suggests public reporting could act as an incentive for quality improvement. Although centralization of cancer surgical services has been considered a key structural determinant to improving outcomes, our results suggest further evaluation of the relationship between the organization of services and outcomes is imperative. In this regard, the empirical framework we have used in this study provides a template for policy makers to develop a robust evidence base to informing the optimum configuration of cancer services to drive equality and outcomes.

## AUTHOR CONTRIBUTIONS

**Lu Han**: Data curation, formal analysis, project administration, visualization, and writing–original draft. **Jemma M. Boyle**: Data curation, formal analysis, writing–original draft, and writing–review and editing. **Kate Walker**: Methodology, project administration, supervision, and writing–review and editing. **Angela Kuryba**: Data curation, formal analysis, and writing–review. **Michael S. Braun**: Writing–review and editing. **Nicola Fearnhead**: Writing–review and editing. **David Jayne**: Conceptualization and writing–review and editing. **Richard Sullivan:** Conceptualization, validation, writing–original draft, and writing–review and editing. **Jan van der Meulen**: Conceptualization, methodology, supervision, writing–original draft, and writing–review and editing. **Ajay Aggarwal**: Conceptualization, data curation, funding acquisition, methodology, project administration, resources, supervision, writing–original draft, and writing–review and editing.

## CONFLICTS OF INTEREST

Michael S. Braun reports fees from Pierre Fabre Pharmaceuticals, Inc. The other authors made no disclosures.

## Supporting information

Supplementary Material S1
