# Supplementary material for: Impact of patient choice and hospital competition on patient outcomes after rectal cancer surgery: A national population‐based study
Source: Cancer. 2022 Oct 19;129(1):130–41. doi: 10.1002/cncr.34504 (PMC10092598; doi:10.1002/cncr.34504)
Supplement: Supplementary file 1 — Supplementary Material S1 [file CNCR-129-130-s001.docx]

**Appendix Table 1. Patient characteristics according to the competitiveness of the hospital environment (measured by the Spatial Competition Index (SCI)).**

|  | **SCI^a^ - 1st quartile** | | **SCI - 2nd quartile** | | **SCI - 3rd quartile** | | **SCI - 4th quartile** | | **Pearson's χ^2^ test** |
| --- | --- | --- | --- | --- | --- | --- | --- | --- | --- |
|  | **n** | **%** | **n** | **%** | **n** | **%** | **n** | **%** |  |
| **No. of patients** | **3,350** | **100** | **3,598** | **100** | **3,198** | **100** | **1,837** | **100** |  |
| **Age** – mean (SD) | 68 (10.7) | | 67 (11.2) | | 66 (11.1) | | 65 (12.2) | |  |
| <50 | 187 | 5.6 | 245 | 6.8 | 218 | 6.8 | 189 | 10.3 | p<0.001 |
| 50-59 | 544 | 16.2 | 631 | 17.5 | 592 | 18.5 | 361 | 19.7 |  |
| 60-74 | 1,684 | 50.3 | 1,811 | 50.3 | 1,619 | 50.6 | 874 | 47.6 |  |
| 75-84 | 820 | 24.5 | 797 | 22.2 | 680 | 21.3 | 362 | 19.7 |  |
| >=85 | 115 | 3.4 | 114 | 3.2 | 89 | 2.8 | 51 | 2.8 |  |
| **Sex** |  |  |  |  |  |  |  |  |  |
| Male | 2,181 | 65.1 | 2,366 | 65.8 | 2,072 | 64.8 | 1,186 | 64.6 | p=0.79 |
| Female | 1,169 | 34.9 | 1,232 | 34.2 | 1,126 | 35.2 | 651 | 35.4 |  |
| **Ethnicity** |  |  |  |  |  |  |  |  |  |
| White | 3,142 | 93.8 | 3,299 | 91.7 | 2,893 | 90.5 | 1,507 | 82.0 | p<0.001 |
| Mixed | 46 | 1.4 | 102 | 2.8 | 123 | 3.9 | 266 | 14.5 |  |
| Missing | 162 | 4.8 | 197 | 5.5 | 182 | 5.7 | 64 | 3.5 |  |
| **Socioeconomic deprivation (IMD quintiles)** |  |  |  |  |  |  |  |  |  |
| 1^st^ (least deprived) | 338 | 10.1 | 441 | 12.3 | 682 | 21.3 | 316 | 17.2 | p<0.001 |
| 2^nd^ | 637 | 19.0 | 498 | 13.8 | 544 | 17.0 | 382 | 20.8 |  |
| 3^rd^ | 850 | 25.4 | 769 | 21.4 | 576 | 18.0 | 375 | 20.4 |  |
| 4^th^ | 848 | 25.3 | 918 | 25.5 | 608 | 19.0 | 365 | 19.9 |  |
| 5^th^ | 675 | 20.2 | 963 | 26.8 | 780 | 24.4 | 395 | 21.5 |  |
| Missing | 2 | 0.1 | 9 | 0.3 | 8 | 0.3 | 4 | 0.2 |  |
| **Number of comorbidities** |  |  |  |  |  |  |  |  |  |
| 0 | 1,950 | 58.2 | 2,133 | 59.3 | 1,921 | 60.1 | 1,062 | 57.8 | p=0.69 |
| 1 | 968 | 28.9 | 1,008 | 28.0 | 873 | 27.3 | 538 | 29.3 |  |
| ≥2 | 432 | 12.9 | 457 | 12.7 | 404 | 12.6 | 237 | 12.9 |  |
| **ASA grade** |  |  |  |  |  |  |  |  |  |
| 1 | 514 | 15.3 | 458 | 12.7 | 479 | 15.0 | 309 | 16.8 | p<0.001 |
| 2 | 1,831 | 54.7 | 2,270 | 63.1 | 1,880 | 58.8 | 1,080 | 58.8 |  |
| ≥3 | 789 | 23.6 | 764 | 21.2 | 684 | 21.4 | 394 | 21.5 |  |
| Missing | 216 | 6.5 | 106 | 3.0 | 155 | 4.9 | 54 | 2.9 |  |
| **Performance status** |  |  |  |  |  |  |  |  |  |
| 0 (Normal activity) | 2,142 | 63.9 | 2,014 | 56.0 | 1,708 | 53.4 | 1,061 | 57.8 | p<0.001 |
| 1 (Walk & light work) | 733 | 21.9 | 884 | 24.6 | 750 | 23.5 | 537 | 29.2 |  |
| 2+ (Walk & all self-care:up >50%) | 173 | 5.2 | 198 | 5.5 | 316 | 9.9 | 170 | 9.3 |  |
| Missing | 302 | 9.0 | 502 | 14.0 | 424 | 13.3 | 69 | 3.8 |  |
| **T staging** |  |  |  |  |  |  |  |  |  |
| T1 | 409 | 12.2 | 466 | 13.0 | 419 | 13.1 | 244 | 13.3 | p<0.001 |
| T2 | 915 | 27.3 | 1,081 | 30.0 | 912 | 28.5 | 508 | 27.7 |  |
| T3 | 1,604 | 47.9 | 1,679 | 46.7 | 1,428 | 44.7 | 840 | 45.7 |  |
| T4 | 179 | 5.3 | 199 | 5.5 | 182 | 5.7 | 120 | 6.5 |  |
| Missing | 243 | 7.3 | 173 | 4.8 | 257 | 8.0 | 125 | 6.8 |  |
| **N staging** |  |  |  |  |  |  |  |  |  |
| N0 | 2,002 | 59.8 | 2,252 | 62.6 | 1,939 | 60.6 | 1,132 | 61.6 | p<0.001 |
| N1 | 790 | 23.6 | 827 | 23.0 | 706 | 22.1 | 403 | 21.9 |  |
| N2 | 317 | 9.5 | 357 | 9.9 | 296 | 9.3 | 164 | 8.9 |  |
| Missing | 241 | 7.2 | 162 | 4.5 | 257 | 8.0 | 138 | 7.5 |  |
| **M staging** |  |  |  |  |  |  |  |  |  |
| M0 | 3,100 | 92.5 | 3,420 | 95.1 | 2,952 | 92.3 | 1,695 | 92.3 | p<0.001 |
| M1 | 23 | 0.7 | 29 | 0.8 | 28 | 0.9 | 25 | 1.4 |  |
| Missing | 227 | 6.8 | 149 | 4.1 | 218 | 6.8 | 117 | 6.4 |  |
| Notes  SCI – spatial competition index is a hospital-based measure assessing the level of competition between hospitals within a 30-minute drive time.  This has been categorized into four quartiles based on the level of competition. 1^st^ quartile represent hospitals in the lowest competition areas  and 4^th^ quartile represents hospitals in the highest competition areas*.* | | | | | | | | | |

**Appendix Table 2. Patient characteristics according to whether the hospital was a successful or unsuccessful competitor.**

|  | **Successful Competitor** | | **Unsuccessful Competitor** | | **Hospitals with no significant gain**  **or loss of patients** | | **Pearson's χ^2^ test** |
| --- | --- | --- | --- | --- | --- | --- | --- |
|  | **n** | **%** | **n** | **%** | **n** | **%** |  |
| **No. of patients** | **4,314** | **100** | **3,403** | **100** | **4,266** | **100** |  |
| **Age** - mean (SD) | 66 (11.5) | | 67 (11.1) | | 67 (11.0) | |  |
| <50 | 323 | 7.5 | 229 | 6.7 | 287 | 6.7 | p=0.43 |
| 50-59 | 770 | 17.9 | 594 | 17.5 | 764 | 17.9 |  |
| 60-74 | 2,117 | 49.1 | 1,713 | 50.3 | 2,158 | 50.6 |  |
| 75-84 | 975 | 22.6 | 772 | 22.7 | 912 | 21.4 |  |
| >=85 | 129 | 3.0 | 95 | 2.8 | 145 | 3.4 |  |
| **Sex** |  |  |  |  |  |  |  |
| Male | 2,824 | 65.5 | 2,211 | 65.0 | 2,770 | 64.9 | p=0.85 |
| Female | 1,490 | 34.5 | 1,192 | 35.0 | 1,496 | 35.1 |  |
| **Ethnicity** |  |  |  |  |  |  |  |
| White | 3,858 | 89.4 | 3,026 | 88.9 | 3,957 | 92.8 | p<0.001 |
| Mixed | 234 | 5.4 | 195 | 5.7 | 108 | 2.5 |  |
| Missing | 222 | 5.2 | 182 | 5.4 | 201 | 4.7 |  |
| **Socioeconomic deprivation (IMD quintiles)** |  |  |  |  |  |  |  |
| 1^st^ (least deprived) | 572 | 13.3 | 664 | 19.5 | 541 | 12.7 | p<0.001 |
| 2^nd^ | 667 | 15.5 | 652 | 19.2 | 742 | 17.4 |  |
| 3^rd^ | 983 | 22.8 | 694 | 20.4 | 893 | 20.9 |  |
| 4^th^ | 1,037 | 24.0 | 748 | 22.0 | 954 | 22.4 |  |
| 5^th^ | 1,049 | 24.3 | 636 | 18.7 | 1,128 | 26.4 |  |
| Missing | 6 | 0.1 | 9 | 0.3 | 8 | 0.2 |  |
| **Number of comorbidities** |  |  |  |  |  |  |  |
| 0 | 2,560 | 59.3 | 2,014 | 59.2 | 2,492 | 58.4 | p=0.46 |
| 1 | 1,228 | 28.5 | 934 | 27.5 | 1,225 | 28.7 |  |
| ≥2 | 526 | 12.2 | 455 | 13.4 | 549 | 12.9 |  |
| **ASA grade** |  |  |  |  |  |  |  |
| 1 | 676 | 15.7 | 423 | 12.4 | 661 | 15.5 | p<0.001 |
| 2 | 2,540 | 58.9 | 1,999 | 58.7 | 2,522 | 59.1 |  |
| ≥3 | 891 | 20.7 | 835 | 24.5 | 905 | 21.2 |  |
| Missing | 207 | 4.8 | 146 | 4.3 | 178 | 4.2 |  |
| **Performance status** |  |  |  |  |  |  |  |
| 0 (Normal activity) | 2,601 | 60.3 | 1,865 | 54.8 | 2,459 | 57.6 | p<0.001 |
| 1 (Walk & light work) | 1,029 | 23.9 | 830 | 24.4 | 1,045 | 24.5 |  |
| 2+ (Walk & all self-care:up >50%) | 289 | 6.7 | 298 | 8.8 | 270 | 6.3 |  |
| Missing | 395 | 9.2 | 410 | 12.1 | 492 | 11.5 |  |
| **T staging** |  |  |  |  |  |  |  |
| T1 | 570 | 13.2 | 403 | 11.8 | 565 | 13.2 | p=0.02 |
| T2 | 1,221 | 28.3 | 985 | 29.0 | 1,210 | 28.4 |  |
| T3 | 2,029 | 47.0 | 1,585 | 46.6 | 1,937 | 45.4 |  |
| T4 | 253 | 5.9 | 193 | 5.7 | 234 | 5.5 |  |
| Missing | 241 | 5.6 | 237 | 7.0 | 320 | 7.5 |  |
| **N staging** |  |  |  |  |  |  |  |
| N0 | 2,649 | 61.4 | 2,063 | 60.6 | 2,613 | 61.3 | p=0.01 |
| N1 | 996 | 23.1 | 774 | 22.7 | 956 | 22.4 |  |
| N2 | 429 | 9.9 | 332 | 9.8 | 373 | 8.7 |  |
| Missing | 240 | 5.6 | 234 | 6.9 | 324 | 7.6 |  |
| **M staging** |  |  |  |  |  |  |  |
| M0 | 4,073 | 94.4 | 3,155 | 92.7 | 3,939 | 92.3 | p=0.001 |
| M1 | 38 | 0.9 | 27 | 0.8 | 40 | 0.9 |  |
| Missing | 203 | 4.7 | 221 | 6.5 | 287 | 6.7 |  |

Notes

Successful competitors are centres that have a statistically significant net gain of patients and Unsuccessful competitors are centers that had a statistically significant net loss of patients (See Methods).

**Appendix Table 3. Association between the competitiveness of the hospital environment, (measured by the Spatial Competition Index)**

**and the odds of undergoing a primary rectal cancer procedure with permanent stoma formation**.

|  | **Stoma (primary intention)^a^** | | | **Stoma (primary intention)** | | |
| --- | --- | --- | --- | --- | --- | --- |
|  | OR**^b^** | 95% CI | *p* | OR**^c^** | 95% CI | *p* |
| **Hospital-level characteristics** |  |  |  |  |  |  |
| SCI**^d^** 1^st^ quartile | Ref |  |  | Ref |  |  |
| 2nd quartile | 0.78 | [0.61 - 0.99] | 0.04 | 0.76 | [0.58 - 0.98] | 0.03 |
| 3rd quartile | 0.76 | [0.60 - 0.95] | 0.02 | 0.74 | [0.58 - 0.93] | 0.01 |
| 4th quartile | 0.73 | [0.55 - 0.97] | 0.03 | 0.78 | [0.59 - 1.05] | 0.10 |
| Volume ( ref: 1st tertile - low) |  |  |  |  |  |  |
| 2nd tertile - medium |  |  |  | 1.21 | [0.93 - 1.58] | 0.15 |
| 3rd tertile - high |  |  |  | 1.29 | [1.02 - 1.63] | 0.03 |
| **Patient-level characteristics** |  |  |  |  |  |  |
| Age (ref: <50) |  |  |  |  |  |  |
| 50-59 | 1.06 | [0.88 - 1.28] | 0.53 | 1.06 | [0.88 - 1.28] | 0.53 |
| 60-74 | 1.19 | [1.00 - 1.41] | 0.05 | 1.19 | [1.00 - 1.41] | 0.05 |
| 75-84 | 1.80 | [1.50 - 2.15] | <0.001 | 1.80 | [1.50 - 2.16] | <0.001 |
| >=85 | 3.37 | [2.50 - 4.53] | <0.001 | 3.37 | [2.50 - 4.54] | <0.001 |
| Sex - Female (ref: Male) | 1.02 | [0.92 - 1.12] | 0.75 | 1.02 | [0.92 - 1.12] | 0.76 |
| Ethnicity - non-white (ref: White) | 0.75 | [0.61 - 0.93] | 0.01 | 0.75 | [0.61 - 0.93] | 0.01 |
| Socioeconomic deprivation (IMD quintile; ref: 1st - least deprived) |  |  |  |  |  |  |
| 2nd | 1.03 | [0.90 - 1.18] | 0.69 | 1.03 | [0.90 - 1.18] | 0.69 |
| 3rd | 0.94 | [0.80 - 1.10] | 0.42 | 0.94 | [0.80 - 1.10] | 0.41 |
| 4th | 0.91 | [0.79 - 1.04] | 0.17 | 0.91 | [0.79 - 1.04] | 0.17 |
| 5th | 0.79 | [0.68 - 0.91] | 0.002 | 0.78 | [0.67 - 0.91] | 0.002 |
| Number of comorbidities (ref: none) |  |  |  |  |  |  |
| 1 | 1.13 | [1.02 - 1.25] | 0.02 | 1.13 | [1.02 - 1.25] | 0.02 |
| ≥2 | 1.23 | [1.07 - 1.40] | 0.003 | 1.22 | [1.07 - 1.40] | 0.003 |
| ASA grade (ref: 1) |  |  |  |  |  |  |
| 2 | 1.20 | [1.04 - 1.38] | 0.01 | 1.20 | [1.04 - 1.38] | 0.01 |
| ≥3 | 1.56 | [1.31 - 1.85] | <0.001 | 1.56 | [1.31 - 1.85] | <0.001 |
| Performance status (ref: 0-Normal activity) |  |  |  |  |  |  |
| 1 (Walk & light work) | 1.14 | [1.03 - 1.27] | 0.01 | 1.14 | [1.03 - 1.27] | 0.02 |
| 2+ (Walk & all self care:up >50%) | 1.33 | [1.12 - 1.57] | 0.001 | 1.33 | [1.12 - 1.57] | 0.001 |

**Appendix Table 3. Association between the competitiveness of the hospital environment, (measured by the Spatial Competition Index)**

**and the odds of undergoing a primary rectal cancer procedure with permanent stoma formation  *(continued)***

| T staging (ref: T1) |  |  |  |  |  |  |  |  |  |  |  |  |
| --- | --- | --- | --- | --- | --- | --- | --- | --- | --- | --- | --- | --- |
| T2 | 1.22 | [1.05 - 1.42] | 0.01 | 1.22 | [1.05 - 1.42] |  |  |  |  |  |  |  |
| T3 | 1.00 | [0.86 - 1.15] | 0.95 | 1.00 | [0.87 - 1.15] |  |  |  |  |  |  |  |
| T4 | 1.33 | [1.07 - 1.66] | 0.01 | 1.33 | [1.07 - 1.65] |  |  |  |  |  |  |  |
| N staging (ref: N0) |  |  |  |  |  |  |  |  |  |  |  |  |
| N1 | 0.95 | [0.86 - 1.05] | 0.28 | 0.95 | [0.86 - 1.05] |  |  |  |  |  |  |  |
| N2 | 0.92 | [0.79 - 1.07] | 0.30 | 0.92 | [0.79 - 1.07] |  |  |  |  |  |  |  |
| M staging (ref: M0) |  |  |  |  |  |  |  |  |  |  |  |  |
| M1 | 1.03 | [0.69 - 1.55] | 0.89 | 1.03 | [0.69 - 1.55] |  |  |  |  |  |  |  |
| Pre-operative radiotherapy (ref: no treatment) |  |  |  |  |  |  |  |  |  |  |  |  |
| Long Course RT pre surgery | 3.72 | [3.23 - 4.28] | <0.001 | 3.72 | [3.23 - 4.28] |  |  |  |  |  |  |  |
| Short Course RT pre surgery | 2.16 | [1.77 - 2.63] | <0.001 | 2.16 | [1.77 - 2.64] |  |  |  |  |  |  |  |
| Year of surgery (ref: 2016) |  |  |  |  |  |  |  |  |  |  |  |  |
| 2017 | 1.05 | [0.94 - 1.18] | 0.42 | 1.05 | [0.93 - 1.18] |  |  |  |  |  |  |  |
| 2018 | 1.06 | [0.94 - 1.20] | 0.36 | 1.06 | [0.93 - 1.20] |  |  |  |  |  |  |  |
| 2019 | 1.08 | [0.94 - 1.25] | 0.28 | 1.08 | [0.94 - 1.25] |  |  |  |  |  |  |  |
|  |  |  |  |  |  |  |  |  |  |  |  |  |
| Variance of constant by sites | 1.31 | [1.19 - 1.45] | <0.001 | 1.31 | [1.19 - 1.44] |  |  |  |  |  |  |  |
| Constant | 0.21 | [0.15 - 0.29] | <0.001 | 0.18 | [0.13 - 0.26] |  |  |  |  |  |  |  |
|  |  |  |  |  |  |  |  |  |  |  |  |  |
| Observations | 11,983 |  |  | 11,983 |  |  |  |  |  |  |  |  |
| Notes  Robust 95% CI in brackets. |  |  |  |  |  |  |  |  |  |  |  |  |
| 1. This included patients who had an APR, Hartmann’s or Pelvic exenteration. 2. Odds ratio adjusted for patient-level characteristics (excluding procedure volume) 3. Odds ratio adjusted for patient-level characteristics (including procedure volume) 4. SCI – spatial competition index is a hospital-based measure assessing the level of   competition between hospitals within a 30-minute drive time. This has been categorized  into four quartiles based on the level of competition. 1^st^ quartile represent hospitals in the  lowest competition areas and 4^th^ quartile represents hospitals in the highest competition areas*.* | | | |  |  |  |  |  |  |  |  |  |
|  | | | | | |  |  |  |  |  |  |  |

**Appendix Table 4. Association between whether a hospital was a successful or unsuccessful competitor and the odds of a 90-day readmission.**

|  | **90-day readmission** | | | **90-day readmission** | | |
| --- | --- | --- | --- | --- | --- | --- |
|  | OR**^a^** | 95% CI | *p* | OR**^b^** | 95% CI | *p* |
| **Hospital-level characteristics** |  |  |  |  |  |  |
| Unsuccessful competitors**^c^** | Ref |  |  | Ref |  |  |
| Successful competitors | 0.86 | [0.76 - 0.97] | 0.01 | 0.85 | [0.74 - 0.97] | 0.02 |
| Hospitals with no significant gain or loss of patients | 0.88 | [0.78 - 0.99] | 0.03 | 0.88 | [0.78 - 0.99] | 0.03 |
| Volume ( ref: 1st tertile - low) |  |  |  |  |  |  |
| 2nd tertile - medium |  |  |  | 1.00 | [0.86 - 1.15] | 0.96 |
| 3rd tertile - high |  |  |  | 1.02 | [0.89 - 1.17] | 0.79 |
| **Patient-level characteristics** |  |  |  |  |  |  |
| Age (ref: <50) |  |  |  |  |  |  |
| 50-59 | 0.68 | [0.57 - 0.81] | <0.001 | 0.68 | [0.57 - 0.81] | <0.001 |
| 60-74 | 0.66 | [0.56 - 0.78] | <0.001 | 0.66 | [0.56 - 0.78] | <0.001 |
| 75-84 | 0.60 | [0.50 - 0.72] | <0.001 | 0.60 | [0.50 - 0.72] | <0.001 |
| >=85 | 0.43 | [0.32 - 0.58] | <0.001 | 0.43 | [0.32 - 0.58] | <0.001 |
| Sex - Female (ref: Male) | 0.89 | [0.81 - 0.97] | 0.01 | 0.89 | [0.81 - 0.97] | 0.01 |
| Ethnicity - non-white (ref: White) | 1.23 | [1.01 - 1.49] | 0.04 | 1.23 | [1.01 - 1.49] | 0.04 |
| Socioeconomic deprivation (IMD quintile; ref: 1st - least deprived) |  |  |  |  |  |  |
| 2nd | 1.22 | [1.07 - 1.40] | 0.003 | 1.22 | [1.07 - 1.40] | 0.003 |
| 3rd | 1.07 | [0.94 - 1.21] | 0.32 | 1.07 | [0.94 - 1.21] | 0.32 |
| 4th | 1.26 | [1.11 - 1.44] | <0.001 | 1.26 | [1.11 - 1.44] | <0.001 |
| 5th | 1.10 | [0.97 - 1.24] | 0.15 | 1.10 | [0.97 - 1.24] | 0.16 |
| Number of comorbidities (ref: none) |  |  |  |  |  |  |
| 1 | 1.04 | [0.95 - 1.14] | 0.43 | 1.04 | [0.95 - 1.14] | 0.43 |
| ≥2 | 1.50 | [1.32 - 1.71] | <0.001 | 1.50 | [1.32 - 1.71] | <0.001 |
| ASA grade (ref: 1) |  |  |  |  |  |  |
| 2 | 1.06 | [0.93 - 1.21] | 0.40 | 1.06 | [0.93 - 1.21] | 0.41 |
| ≥3 | 1.15 | [0.99 - 1.34] | 0.07 | 1.15 | [0.99 - 1.34] | 0.07 |
| Performance status (ref: 0-Normal activity) |  |  |  |  |  |  |
| 1 (Walk & light work) | 0.98 | [0.88 - 1.10] | 0.72 | 0.98 | [0.88 - 1.09] | 0.72 |
| 2+ (Walk & all self care:up >50%) | 1.04 | [0.89 - 1.22] | 0.62 | 1.04 | [0.89 - 1.22] | 0.63 |

**Appendix Table 4. Association between whether a hospital was a successful or unsuccessful competitor and the odds of a 90-day readmission (continued).**

| T staging (ref: T1) |  | |  | |  |  | |  | |  |  |  |  |  |  |  |
| --- | --- | --- | --- | --- | --- | --- | --- | --- | --- | --- | --- | --- | --- | --- | --- | --- |
| T2 | 1.17 | | [0.96 - 1.42] | | 0.13 | 1.17 | | [0.96 - 1.42] | | 0.13 |  |  |  |  |  |  |
| T3 | 1.19 | | [0.99 - 1.43] | | 0.07 | 1.19 | | [0.99 - 1.43] | | 0.07 |  |  |  |  |  |  |
| T4 | 0.97 | | [0.71 - 1.31] | | 0.82 | 0.97 | | [0.71 - 1.31] | | 0.82 |  |  |  |  |  |  |
| N staging (ref: N0) |  | |  | |  |  | |  | |  |  |  |  |  |  |  |
| N1 | 0.84 | | [0.73 - 0.96] | | 0.01 | 0.84 | | [0.73 - 0.96] | | 0.01 |  |  |  |  |  |  |
| N2 | 1.02 | | [0.83 - 1.25] | | 0.84 | 1.02 | | [0.83 - 1.25] | | 0.84 |  |  |  |  |  |  |
| M staging (ref: M0) |  | |  | |  |  | |  | |  |  |  |  |  |  |  |
| M1 | 1.19 | | [0.70 - 2.02] | | 0.52 | 1.19 | | [0.70 - 2.02] | | 0.52 |  |  |  |  |  |  |
| Pre-operative radiotherapy (ref: no treatment) |  | |  | |  |  | |  | |  |  |  |  |  |  |  |
| Long Course RT pre surgery | 1.20 | | [1.07 - 1.35] | | 0.003 | 1.20 | | [1.07 - 1.35] | | 0.003 |  |  |  |  |  |  |
| Short Course RT pre surgery | 1.00 | | [0.82 - 1.23] | | 0.98 | 1.00 | | [0.82 - 1.23] | | 0.98 |  |  |  |  |  |  |
| Year of surgery (ref: 2016) |  | |  | |  |  | |  | |  |  |  |  |  |  |  |
| 2017 | 1.04 | | [0.89 - 1.21] | | 0.62 | 1.04 | | [0.89 - 1.21] | | 0.62 |  |  |  |  |  |  |
| 2018 | 1.16 | | [1.00 - 1.35] | | 0.06 | 1.16 | | [0.99 - 1.35] | | 0.06 |  |  |  |  |  |  |
| 2019 | 1.14 | | [0.97 - 1.35] | | 0.11 | 1.14 | | [0.97 - 1.35] | | 0.11 |  |  |  |  |  |  |
|  |  | |  | |  |  | |  | |  |  |  |  |  |  |  |
| Variance of constant by sites | 1.07 | | [1.03 - 1.11] | | <0.001 | 1.07 | | [1.03 - 1.11] | | <0.001 |  |  |  |  |  |  |
| Constant | 0.18 | | [0.13 - 0.25] | | <0.001 | 0.18 | | [0.13 - 0.25] | | <0.001 |  |  |  |  |  |  |
|  |  | |  | |  |  | |  | |  |  |  |  |  |  |  |
| Observations | 11,983 | |  | |  | 11,983 | |  | |  |  |  |  |  |  |  |
| Notes |  | |  | |  |  | |  | |  |  |  |  |  |  |  |
| Robust 95% CI in brackets. | |  | |  | | |  | |  |  |  |  |  |  |  |  |
| 1. Odds ratio adjusted for patient-level characteristics (excluding procedure volume) 2. Odds ratio adjusted for patient-level characteristics (including procedure volume) 3. Successful competitors are centres that have a statistically significant net gain   of patients and Unsuccessful competitors are centers that had a statistically  significant net loss of patients (See Methods).  *.* | | | | | | | | |  |  |  |  |  |  |  |  |

**Appendix Table 5. Association between whether a hospital was a successful or unsuccessful competitor and**

**the odds of having a persistent stoma 18 months after anterior resection.**

|  | **Stoma (at 18 months)** | | | **Stoma (at 18 months)** | | |
| --- | --- | --- | --- | --- | --- | --- |
|  | OR**^a^** | 95% CI | *p* | OR**^b^** | 95% CI | *p* |
| **Hospital-level characteristics** |  |  |  |  |  |  |
| Unsuccessful competitors**^c^** | Ref |  |  | Ref |  |  |
| Successful competitors | 0.75 | [0.61 - 0.93] | 0.01 | 0.71 | [0.58 - 0.88] | 0.002 |
| Hospitals with no significant gain or loss of patients | 0.96 | [0.75 - 1.23] | 0.73 | 0.93 | [0.73 - 1.19] | 0.57 |
| Volume (ref: 1st tertile - low) |  |  |  |  |  |  |
| 2nd tertile - medium |  |  |  | 1.12 | [0.86 - 1.44] | 0.41 |
| 3rd tertile - high |  |  |  | 1.18 | [0.92 - 1.50] | 0.19 |
| **Patient-level characteristics** |  |  |  |  |  |  |
| Age (ref: <50) |  |  |  |  |  |  |
| 50-59 | 1.15 | [0.86 - 1.53] | 0.36 | 1.14 | [0.86 - 1.53] | 0.36 |
| 60-74 | 1.28 | [0.95 - 1.72] | 0.11 | 1.27 | [0.95 - 1.72] | 0.11 |
| 75-84 | 1.80 | [1.31 - 2.48] | <0.001 | 1.80 | [1.31 - 2.48] | <0.001 |
| >=85 | 3.74 | [2.35 - 5.94] | <0.001 | 3.74 | [2.35 - 5.95] | <0.001 |
| Sex - Female (ref: Male) | 0.81 | [0.71 - 0.92] | 0.001 | 0.81 | [0.71 - 0.92] | 0.001 |
| Ethnicity - non-white (ref: White) | 1.06 | [0.80 - 1.42] | 0.68 | 1.07 | [0.81 - 1.43] | 0.63 |
| IMD quintile (ref: 1st - least deprived) |  |  |  |  |  |  |
| 2nd | 0.79 | [0.64 - 0.97] | 0.02 | 0.79 | [0.64 - 0.97] | 0.02 |
| 3rd | 0.74 | [0.62 - 0.88] | 0.001 | 0.74 | [0.62 - 0.88] | 0.001 |
| 4th | 0.82 | [0.68 - 0.99] | 0.04 | 0.82 | [0.68 - 0.99] | 0.04 |
| 5th | 0.69 | [0.57 - 0.83] | <0.001 | 0.69 | [0.57 - 0.83] | <0.001 |
| Charlson comorbidity (ref: none) |  |  |  |  |  |  |
| 1 | 1.14 | [1.00 - 1.30] | 0.06 | 1.14 | [1.00 - 1.31] | 0.05 |
| ≥2 | 1.55 | [1.26 - 1.91] | <0.001 | 1.55 | [1.26 - 1.90] | <0.001 |
| ASA grade (ref: 1) |  |  |  |  |  |  |
| 2 | 1.04 | [0.86 - 1.26] | 0.68 | 1.04 | [0.86 - 1.26] | 0.70 |
| ≥3 | 1.38 | [1.08 - 1.76] | 0.01 | 1.38 | [1.08 - 1.76] | 0.01 |
| Performance status (ref: 0-Normal activity) |  |  |  |  |  |  |
| 1 (Walk & light work) | 1.11 | [0.95 - 1.29] | 0.19 | 1.11 | [0.95 - 1.29] | 0.19 |
| 2+ (Walk & all self care:up >50%) | 1.47 | [1.16 - 1.88] | 0.002 | 1.48 | [1.16 - 1.88] | 0.002 |

**Appendix Table 5. Appendix Table 5. Association between whether a hospital was a successful or unsuccessful competitor and**

**the odds of having a persistent stoma 18 months after anterior resection (continued).**

| T staging (ref: T1) |  | |  | | |  | |  | |  | |  | |  |  |  |  |  |  |
| --- | --- | --- | --- | --- | --- | --- | --- | --- | --- | --- | --- | --- | --- | --- | --- | --- | --- | --- | --- |
| T2 | 1.30 | [1.05 - 1.60] | | | 0.02 | | 1.30 | | [1.05 - 1.60] | | 0.02 | |  |  |  |  |  |  |  |
| T3 | 1.31 | [1.07 - 1.61] | | | 0.01 | | 1.31 | | [1.07 - 1.61] | | 0.01 | |  |  |  |  |  |  |  |
| T4 | 1.47 | [1.08 – 2.00] | | | 0.02 | | 1.47 | | [1.08 - 1.20] | | 0.02 | |  |  |  |  |  |  |  |
| N staging (ref: N0) |  |  | | |  | |  | |  | |  | |  |  |  |  |  |  |  |
| N1 | 1.30 | [1.13 - 1.50] | | | <0.001 | | 1.30 | | [1.13 - 1.50] | | <0.001 | |  |  |  |  |  |  |  |
| N2 | 1.55 | [1.28 - 1.88] | | | <0.001 | | 1.55 | | [1.28 - 1.88] | | <0.001 | |  |  |  |  |  |  |  |
| M staging (ref: M0) |  |  | | |  | |  | |  | |  | |  |  |  |  |  |  |  |
| M1 | 0.98 | [0.52 - 1.85] | | | 0.95 | | 0.98 | | [0.52 - 1.86] | | 0.96 | |  |  |  |  |  |  |  |
| Pre-operative radiotherapy (ref: no treatment) |  |  | | |  | |  | |  | |  | |  |  |  |  |  |  |  |
| Long Course RT pre surgery | 1.64 | [1.41 - 1.92] | | | <0.001 | | 1.65 | | [1.41 - 1.92] | | <0.001 | |  |  |  |  |  |  |  |
| Short Course RT pre surgery | 1.84 | [1.48 - 2.27] | | | <0.001 | | 1.84 | | [1.48 - 2.28] | | <0.001 | |  |  |  |  |  |  |  |
| Year of surgery (ref: 2016) |  |  | | |  | |  | |  | |  | |  |  |  |  |  |  |  |
| 2017 | 0.95 | [0.81 - 1.11] | | | 0.48 | | 0.94 | | [0.81 - 1.10] | | 0.47 | |  |  |  |  |  |  |  |
| 2018 | 0.93 | [0.79 - 1.10] | | | 0.40 | | 0.93 | | [0.79 - 1.10] | | 0.39 | |  |  |  |  |  |  |  |
| 2019 | 1.07 | [0.87 - 1.31] | | | 0.52 | | 1.07 | | [0.87 - 1.31] | | 0.55 | |  |  |  |  |  |  |  |
|  |  |  | | |  | |  | |  | |  | |  |  |  |  |  |  |  |
| Variance of constant by sites | 1.24 | [1.12 - 1.37] | | | <0.001 | | 1.24 | | [1.12 - 1.36] | | <0.001 | |  |  |  |  |  |  |  |
| Constant | 0.19 | [0.13 - 0.28] | | | <0.001 | | 0.18 | | [0.12 - 0.27] | | <0.001 | |  |  |  |  |  |  |  |
|  |  |  | | |  | |  | |  | |  | |  |  |  |  |  |  |  |
| Observations | 6,485 |  | | |  | | 6,485 | |  | |  | |  |  |  |  |  |  |  |
| Notes  Robust 95% CI in brackets. | | | |  |  |  |  |  |  |  |  |  |  |  |  |  |  |  |  |
| 1. Odds ratio adjusted for patient-level characteristics (excluding procedure volume) 2. Odds ratio adjusted for patient-level characteristics (including procedure volume) 3. Successful competitors are centres that have a statistically significant net gain   of patients and Unsuccessful competitors are centers that had a statistically  significant net loss of patients (See Methods).  *.* | | | | | | | | | | | | | | | | | | | |

**Appendix Table 6. Association between competitiveness of the hospital environment measured**

**by the Spatial Competition Index (SCI) and volume of surgery between 1^st^ April 2015 and 31^st^ March 2019.**

| **Competitive environment (SCI)** | **Average annual volume** | ***p*** |
| --- | --- | --- |
| 1^st^ quartile (41 sites) | 31.1 | < 0.001 |
| 2^nd^ quartile (41 sites) | 31.5 |  |
| 3^rd^ quartile (41 sites) | 30.8 |  |
| 4^th^ quartile (40 sites) | 20.0 |  |
|  |  |  |
| **Successful competition** | **Average annual volume** | ***p*** |
| Successful competitors (49 sites) | 35.2 | < 0.001 |
| Unsuccessful competitors (56 sites) | 22.1 |  |
| Hospitals with no significant gain or loss of patients (58 sites) | 28.8 |  |

Notes

1. SCI – spatial competition index is a hospital-based measure assessing the level of

competition between hospitals within a 30-minute drive time. This has been categorized

into four quartiles based on the level of competition. 1^st^ quartile represent hospitals in the

lowest competition areas and 4^th^ quartile represents hospitals in the highest competition areas.

1. Successful competitors are centres that have a statistically significant net gain

of patients and Unsuccessful competitors are centers that had a statistically

significant net loss of patients (See Methods).
